# Turning the Tide Against Carbapenem-resistant *Acinetobacter baumannii*: Advancing Care With Sulbactam–Durlobactam

**DOI:** 10.1093/ofid/ofaf618

**Published:** 2025-10-17

**Authors:** Michael Satlin, Ryan K Shields, Antoni Torres, Glenn Tillotson

**Affiliations:** Infectious Diseases Clinical Research Unit, Weill Cornell Medicine, New York City, New York, USA; Antibiotic Management Program, Center for Innovative Antimicrobial Therapeutics, University of Pittsburgh, Pennsylvania, USA; Respiratory Critical Care Hospital Clinic of Barcelona, University of Barcelona, Barcelona, Spain; Microbiology Department, GST Micro LLC North, Virginia, USA

**Keywords:** *Acinetobacter baumannii*, carbapenem resistance, multidrug resistance, sulbactam–durlobactam, β-lactamase

## INTRODUCTION

*Acinetobacter baumannii* is a Gram-negative opportunistic pathogen that presents significant challenges in healthcare settings, particularly in intensive care units (ICUs) and long-term acute care hospitals (LTACHs). It is among the leading nosocomial pathogens associated with ventilator-associated pneumonia (VAP), ranking as the 10th most frequently reported pathogen in ICUs, where it accounted for 3.2% of cases. In LTACHs, it plays an even more prominent role, ranking 4th and contributing to 6.6% of VAP cases [1].

The World Health Organization (WHO) has designated carbapenem-resistant *A. baumannii* (CRAB) as a critical priority pathogen due to its extensive antimicrobial resistance, the limited treatment options, and global health impact [2–4].

This paper explores the clinical and epidemiological aspects of *A. baumannii*, the challenges posed by existing therapies, and the emergence of sulbactam–durlobactam (SUL/DUR) as a promising therapeutic option.

## GLOBAL PREVALENCE OF CARBAPENEM RESISTANCE AMONG *A. BAUMANNII* ISOLATES

**Figure 1.**
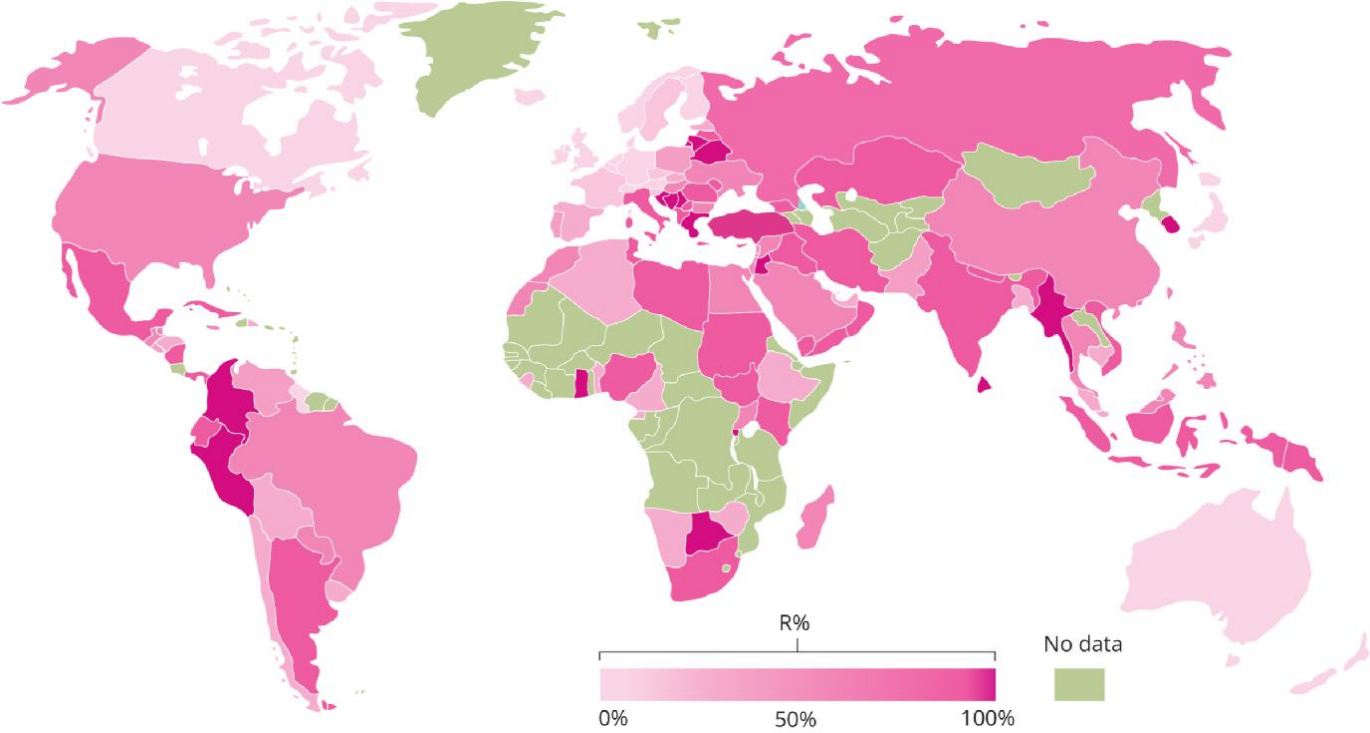
Global percentage of resistance to carbapenems (R%) of *A. baumannii* between 2009 and 2018. Adapted from Ma and McClean [5]. Global percentage of resistance to carbapenems (R%) of *A. baumannii*. The intensity of pink shading indicates estimated carbapenem resistance levels worldwide between 2009 and 2018. Regions shown in green reflect a lack of reliable surveillance or published data. Figure reproduced from [5].

The global epidemiology of *A. baumannii* reflects its position as a leading cause of nosocomial infections and a critical public health challenge due to its rapid acquisition of multidrug resistance (MDR). CRAB is particularly concerning, with its prevalence intensifying in several regions globally. The Mediterranean region remains a significant hotspot, where over 90% of clinical isolates demonstrate carbapenem resistance [5]. Indeed, similar data are reported from various parts of the world as can be seen in Figure 1.

In the United States, surveillance data of healthcare-associated infections from 2015 to 2017 highlight the disproportionate burden of carbapenem resistance among *Acinetobacter spp.*, with carbapenem resistance identified in 41.3% to 52.8% of isolates in patients in ICUs and in 75.4% to 76.9% of isolates from patients in LTACHs [1]. In comparison, *Pseudomonas aeruginosa* exhibited carbapenem resistance ranging from 19.7% to 26.3% in isolates from ICU patients and 35.9% to 61.4% in isolates from LTACH patients, while *Enterobacterales* demonstrated carbapenem resistance rates of 3.5% to 7.2% in ICUs and 9.4% to 13.7% in LTACHs [1]. Additional data from the JMI Laboratories’ SENTRY Antimicrobial Surveillance Program (2014–2021) reinforce these findings, showing that 36.5% of *Acinetobacter baumannii–calcoaceticus* species complex (ACB) isolates were meropenem-resistant [6]. Given these high resistance rates, CRAB infections pose a substantial clinical burden, causing an estimated 8500 infections and 700 deaths [7].

A broader global perspective underscores the growing crisis. Recent modeling by Oldenkamp et al [8], expanding surveillance efforts across multiple regions, found CRAB present in 87% of countries, impacting 99% of the global population. This widespread prevalence disproportionately affects low- and middle-income regions, where healthcare inequities amplify the devastating effects of rising resistance. Figure 1 provides a striking visualization of these trends, mapping key resistance hotspots across the Mediterranean, the Middle East, and Southeast Asia while also highlighting emerging areas of concern, including sub-Saharan Africa.

## MORTALITY AND CLINICAL OUTCOMES OF CRAB INFECTIONS

CRAB infections are associated with significantly higher mortality rates compared with infections caused by carbapenem-susceptible *A. baumannii* (CSAB). A systematic review of 16 studies comparing outcomes between CRAB and CSAB infections reported a 2.22-fold increase in mortality among patients with CRAB infections, with the highest risk observed in individuals with severe underlying conditions or those receiving inappropriate initial treatments [9].

Global mortality data reveal substantial regional differences. A multicenter study found that 30-day mortality among CRAB-infected patients was highest in South-Central America (49%), followed by the United States (25%), China (20%), the Middle East (18%), and Australia–Singapore (6%) [10]. Among infection types, bloodstream infections (BSIs) had the highest 30-day mortality (42%), followed by respiratory infections (23%), wound infections (11%), and urinary tract infections (UTI; 11%).

Several studies have identified key risk factors for mortality after infection due to CRAB. A retrospective cohort study of CRAB infections in ICUs reported a 90-day mortality rate of 30.3%, with higher mortality observed among patients with BSIs and severe comorbidities [11]. Independent risk factors for increased mortality included diabetes, immunocompromised status, high sequential organ failure assessment scores, vasopressor use, and pneumonia as the primary source of bacteremia [12].

A different analysis of 164 patients with CRAB BSIs demonstrated a 30-day mortality rate of 55%, with ST191 identified as the most prevalent strain. Inappropriate empirical therapy was a significant factor associated with mortality [13]. A retrospective cohort study involving 196 patients in a tertiary care teaching hospital reported an in-hospital mortality rate of 58.7%. Predictors of mortality included invasive mechanical ventilation, effective antibiotic treatment lasting ≤6 days, age >58 years, and the absence of coinfection [14].

A U.S. cohort study of 4599 inpatients with *Acinetobacter* infections found that 13.6% died during admission. However, among patients specifically infected with CRAB, the inpatient mortality rate was notably higher at 24.7%, nearly 3 times that of non-CRAB infections (8.5%) [15]. These findings underscore the critical need for early and appropriate therapeutic interventions, particularly in cases of carbapenem-resistant strains.

## ECONOMIC AND HEALTHCARE BURDEN OF CRAB

Beyond its clinical consequences, CRAB infections impose a substantial economic burden on healthcare systems. In the U.S., CRAB infections cost an estimated $281 million annually [7]. A U.S.-based study by Nelson et al [16] estimated the cost of an MDR *A. baumannii* healthcare-associated infection to range from $33 510 to $129,917, depending on the methodology used. In China, patients with CRAB infections incurred $7277 in additional hospital costs and a 15.8-day increase in hospital length of stay compared with those with CSAB infections [17]. Finally, in Europe, the European Centre for Disease Prevention and Control (ECDC) estimated 56 960 CRAB infections in EU/EEA hospitals in 2019, leading to 2749 attributable deaths and 92 991 disability-adjusted life years [18].

## RESISTANCE MECHANISMS IN *ACINETOBACTER BAUMANNII*

*Acinetobacter baumannii* demonstrates remarkable adaptability in its resistance mechanisms, making it a significant challenge in clinical management. One of its primary resistance mechanisms is the production of OXA-type carbapenemases, including OXA-23, OXA-24/40, and OXA-58. Among these, OXA-23 is particularly widespread and is the most common mechanism of carbapenem resistance [10]. Molecular studies have shown that OXA-23 exhibits conformational flexibility near its active site, enabling it to hydrolyze a broad spectrum of β-lactam antibiotics with enhanced catalytic efficiency [19]. Despite its clinical importance, OXA-23 is often underrepresented in commercial molecular diagnostic assays such as BCID2, Verigene, CarbaR, and Carba5, which primarily target carbapenemases like KPC, NDM, and OXA-48 [20]. This omission frequently results in the under detection of OXA-23-producing strains and delays in identifying CRAB. Recent advancements, including the NG-Test DetecTool OXA-23 and RESIST ACINETO assays (currently lacking FDA clearance), have demonstrated high sensitivity and specificity for detecting OXA-23 directly from blood cultures, addressing this gap in diagnostic capability [21–23].

Genome sequencing and in silico analyses have enabled the identification of extended-spectrum β-lactamases (ESBLs) such as *bla*_PER_, *bla*_VEB_, and *bla*_GES_ in *Acinetobacter* spp., of which *bla*_PER_ is most common [24]. The *bla*_PER_ in *Acinetobacter* spp. has been documented from diverse geographic regions, highlighting its global distribution [24]. Of particular concern, the expression of PER-type ESBLs in *A. baumannii* can compromise the activity of newer agents such as cefiderocol.

In addition to carbapenemases and ESBLs, antimicrobial resistance in *A. baumannii* is also mediated by chromosomal class C β-lactamases, known as Acinetobacter-derived cephalosporinases (ADCs). These enzymes confer intrinsic resistance to penicillins and first- and second-generation cephalosporins [24]. Structural variation in ADCs primarily occurs in the Ω-loop region near the cephalosporin binding site, where a common alanine duplication (Adup) has been observed that leads to hydrolysis of third-generation cephalosporins such as cefotaxime and ceftazdime [25]. Three Adup variants of ADC have also demonstrated increased catalytic efficiency against cefiderocol, leading to increases in cefiderocol MIC values. However, most isolates with these ADC variants still demonstrate cefiderocol MICs ≤4 µg/mL [25].

**Figure 2.**
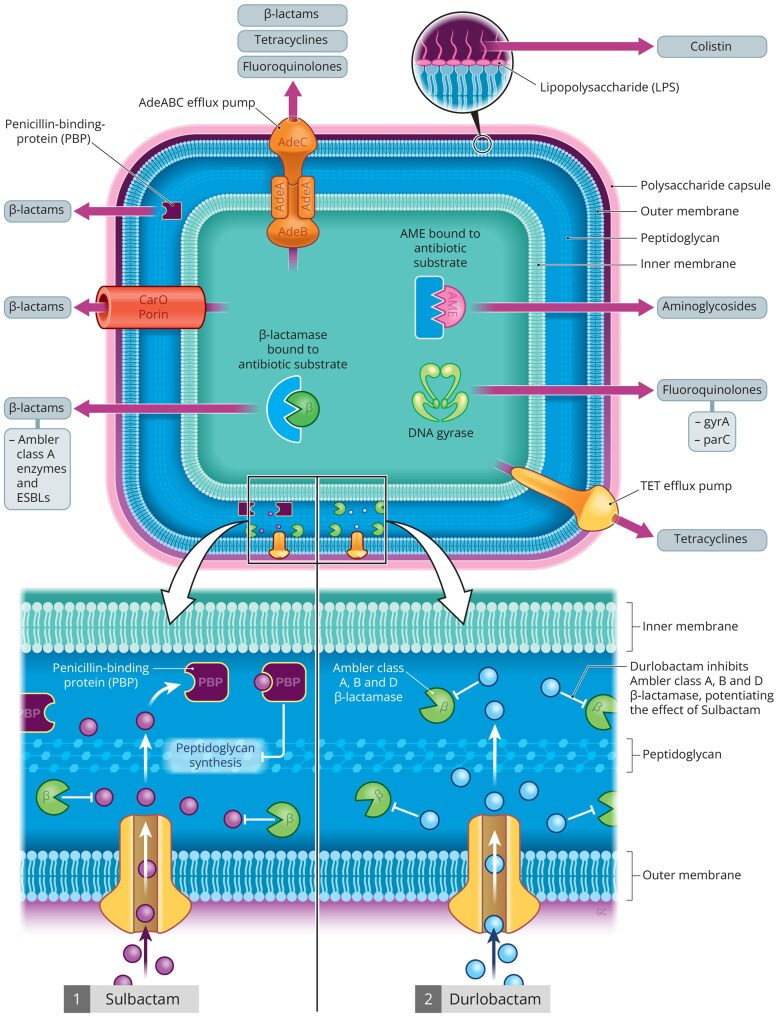
Resistance mechanisms of *Acinetobacter baumannii* to antimicrobial agents. Various resistance mechanisms of *Acinetobacter baumannii* to antimicrobial agents are shown, including the mechanism of action of sulbactam and durlobactam. These include antibiotic-modifying enzymes, efflux pumps, porin alterations, and drug target modifications, along with the antibiotic classes affected by each mechanism. Abbreviations: AMEs, aminoglycoside modifying enzymes; ESBLs, extended-spectrum β-lactamases; LPS, lipopolysaccharide; PBP, penicillin binding protein.

Resistance in *A. baumannii* is further driven by intrinsic mechanisms such as efflux pumps, porin alterations, and chromosomally encoded enzymes such as OXA-51 and AmpC β-lactamases (Figure 2).

Efflux systems, including AdeABC, actively expel antibiotics, reducing concentrations inside the bacterial cell and limiting activity. Modifications to cell wall porins further restrict antibiotic entry, while enzyme degradation by ADCs hydrolyze cephalosporins, diminishing the effectiveness of β-lactam antibiotics [26]. Alterations in penicillin-binding proteins (PBPs), particularly PBP3, also play a critical role in resistance by reducing the binding affinity of β-lactams, including novel agents like cefiderocol [27, 28].

The genomic plasticity of *A. baumannii* exacerbates its resistance profile, enabling the acquisition and dissemination of resistance genes through mobile genetic elements such as transposons and plasmids. High-risk clones, including ST1 and ST2, amplify this problem by spreading resistance determinants via horizontal gene transfer and homologous recombination [2].

Rapid and accurate detection of resistance genes is critical for effective clinical management. Recent innovations in diagnostic tools have shown promise in addressing this need. For instance, a 15-μL recombinase polymerase amplification (RPA) assay demonstrated high sensitivity and specificity in detecting *bla*_OXA-23_ and *bla*_OXA-51_ genes, offering a rapid and cost-effective alternative to traditional PCR and qPCR methods. The RPA assay detected resistance in 90% of *A. baumannii* isolates within 18 minutes, demonstrating its potential as a fast molecular diagnostic tool [29]. However, because the assay was tested on bacterial isolates rather than directly from clinical specimens, its suitability as a point-of-care test remains uncertain.

An increasingly studied feature of *A. baumannii* isolates is its propensity to have heteroresistance to important antibiotics, defined as the presence of a resistant subpopulation within an otherwise susceptible bacterial community. Heteroresistance is not detected by routine antimicrobial susceptibility testing (AST) performed in clinical laboratories [30] and may lead to treatment failure by enabling regrowth of resistant cells during therapy [31]. Heteroresistance is most commonly reported with colistin, a last-resort option for multidrug-resistant *Acinetobacter* infections but has also been described with newer and repurposed agents including cefiderocol and tigecycline [32, 33]. Additional studies are needed to better understand the clinical importance of heteroresistance in *A. baumannii*.

These findings highlight the urgent need for comprehensive surveillance systems, robust diagnostic tools, and innovative therapeutic strategies to combat *A. baumannii* resistance. Targeted diagnostics, such as rapid immunochromatographic assays, combined with novel therapeutic agents that circumvent conventional resistance pathways, represent crucial steps in mitigating the clinical and public health impact of this pathogen. Understanding and addressing the multifaceted resistance mechanisms in *A. baumannii* is essential to curbing its spread and improving patient outcomes.

## CURRENT THERAPIES AND LIMITATIONS

The treatment of CRAB infections remains a significant challenge due to the organism's extensive resistance mechanisms and the limited number of effective therapeutic options. Current therapies differ in pharmacologic profiles and limitations (Table 1), with variable clinical outcomes and mortality rates (Table 2), highlighting the need for improved approaches.

**Table 1.** Overview of Current Therapeutic Options for CRAB

Therapy	Mechanism Of Action	Recommended Dosage	PK/PD Profile	Advantages	Limitations
Ampicillin–Sulbactam [34–38]	Sulbactam acts as a β-lactamase inhibitor and has intrinsic activity against *Acinetobacter* spp., whereas ampicillin alone is inactivated by the organism's intrinsic β-lactamases	9–12 g/day in divided doses (sulbactam component); higher doses may be required for CRAB	Time-dependent killing; sulbactam exhibits bactericidal activity when %fT > MIC ≥ 40%–50%	Effective against certain *Acinetobacter* infections; sulbactam exhibits bactericidal activity via PBP1/PBP3 binding	Resistance due to β-lactamase overexpression; limited efficacy in strains with high-level β-lactamase production; rapid dissemination of resistance genes
Cefiderocol [37, 39–43]	Functions as a siderophore, utilizing bacterial iron transport systems to gain entry and inhibit cell wall synthesis	2 g IV every 8 h (3-h infusion)	Time-dependent killing; %fT > MIC ≥ 75% for efficacy	High activity against carbapenem-resistant strains; stability against various β-lactamases	Limited clinical data; potential for resistance development; higher mortality observed in CRAB-related HABP/VABP in some studies
Eravacycline [44–50]	Binds to the 30S ribosomal subunit to inhibit bacterial protein synthesis by blocking the attachment of aminoacyl-tRNA, thereby preventing elongation of the peptide chain	1 mg/kg IV every 12 h (typically over 60 min); alternatively, 1.5 mg/kg IV every 24 h	Concentration-dependent killing; efficacy is associated with fAUC/MIC ≥ targets of ∼ 28 for stasis and ∼33 for 1-log kill	Broad-spectrum activity including MDR *A. baumannii*; not affected by common tetracycline resistance genes; dose adjustment is not required for renal impairment; favorable safety profile	Limited clinical data; resistance can emerge via efflux pump or regulatory mutations; efficacy in severe CRAB infections unknown; reduced dosage required if used with strong CYP3A4 inducers; hypofibrinogenemia observed in some cases
Minocycline [51, 52]	Inhibits bacterial protein synthesis by binding to the 30S ribosomal subunit, blocking tRNA entry	200 mg IV every 12 h consistent with the CLSI susceptibility breakpoint	Time-dependent killing; efficacy associated with fAUC/MIC ≥12 for stasis and ≥18 for 1-log kill	Effective against MDR and XDR *Acinetobacter*, including some colistin-resistant strains; achieves high tissue concentrations; minimal need for renal adjustment; high oral bioavailability and IV-to-oral switch capability	Limited clinical trial data; clinical experience largely based on retrospective studies; resistance primarily mediated via TetB and efflux pumps; some pharmacokinetic variability
Polymyxins (Colistin, Polymyxin B) [37, 38, 53–55]	Binds to LPS in the outer membrane of Gram-negative bacteria, disrupting membrane integrity	Colistin: 2.5–5 mg/kg/d (based on colistin base activity); Polymyxin B: 1.5–3 mg/kg/d	Concentration-dependent killing; efficacy is associated with AUC/MIC ≥ 50–60 and Cmax/MIC	Broad activity against MDR Gram-negative bacteria; often used for severe infections in combination regimens	Nephrotoxicity and neurotoxicity; increasing resistance rates; limited efficacy in BSI; synergistic combinations often required
SUL/DUR [37, 56–58]	Combines sulbactam's direct antibacterial activity (via PBP1/PBP3 inhibition) with durlobactam's ability to inhibit class A, C, and D β-lactamases	1 g sulbactam/1 g durlobactam IV every 6 h (3-h infusion)	Time-dependent killing; sulbactam requires %fT > MIC ≥ 40% for efficacy; achieves high lung concentrations (∼50% ELF/plasma), supporting its effectiveness for pneumonia caused by CRAB	Demonstrates potent activity against MDR and XDR *Acinetobacter* strains; reduced nephrotoxicity compared with polymyxins	Emerging resistance mechanisms; limited clinical experience; not effective against metallo-β-lactamase-producing strains
Tigecycline [37, 38, 59–62]	Inhibits bacterial protein synthesis by binding to the 30S ribosomal subunit, blocking tRNA entry	Loading dose: 100 mg IV, followed by 50 mg IV every 12 h; High-dose regimen: 100 mg IV every 12 h	Time-dependent killing; efficacy is associated with AUC/MIC ≥ 6–9, but low plasma concentrations limit use in BSIs	Effective against a broad spectrum of bacteria, including MDR strains; useful in soft tissue and intra-abdominal infections	Limited efficacy in BSI; low serum levels necessitate combination regimens; potential for resistance development through efflux pumps

Abbreviations: %fT > MIC, percentage of time the free drug concentration remains above the minimum inhibitory concentration; AUC/MIC, area under the concentration-time curve to minimum inhibitory concentration; BSI, bloodstream infections; ELF, epithelial lining fluid; fAUC/MIC, free area under the concentration-time curve to minimum inhibitory concentration; IV, intravenous; MDR, multidrug-resistant; PD, pharmacodynamics; PK, pharmacokinetics; XDR, extensively drug-resistant.

**Table 2.** Representative Overview of the Efficacy and Outcomes for Select CRAB Therapeutic Options

Therapy	Study Design	Infection Type	Treatment Success Or Failure	Mortality Rate	References
Ampicillin–Sulbactam	Retrospective study	BSI	33% (7/21 clinical failure)	Death during treatment stratified by MIC: ≤ 4 mg/L: 14%, 8 mg/L: 50%, ≥ 16 mg/L: 20% In-hospital mortality stratified by MIC: ≤4 mg/L: 71%, 8 mg/L: 60%, ≥16 mg/L: 20%	[63]
	Prospective cohort study	VAP	23% (3/13 clinical failure) 38.4% (5/13 bacteriological failure)	14-d: 15.3% 28-d: 30.0%	[36]
Cefiderocol	Randomized controlled trial (CREDIBLE-CR)	BSI, HABP, VABP, UTIs	Clinical cure at end of treatment: 66% (53/80) for cefiderocol; 58% (22/38) for BAT Clinical failure at end of treatment: 25% (20/80) for cefiderocol; 40% (15/38) for BAT End of treatment microbiological eradication: 48% (38/80) for cefiderocol; 26% (10/38) for BAT	14-d: 19% for cefiderocol; 12% for BAT 28-d: 25% for cefiderocol; 18% for BAT End-of-study: 34% for cefiderocol; 18% for BAT	[40]
	Randomized controlled trial (APEKS-NP)	HAP, VAP, HCAP	Clinical cure at TOC: 65% (94/145) Microbiological eradication at TOC: 41% (59/145)	14-d: 12.4% 28-d: 21.0% End-of-study: 27%	[64]
Meropenem (high-dose, extended infusion)	Randomized controlled trial (APEKS-NP)	HAP, VAP, HCAP	Clinical cure at TOC: 67% (98/147) Microbiological eradication at TOC: 42% (61/147)	14-d: 11.6% 28-d: 20.5% End-of-study: 23%	[64]
Minocycline	Multicenter observational study	BSI, pneumonia	Clinical response: 90.9% (10/11) overall Monotherapy: 100% (5/5) Combination: 83.3% (5/6) Microbiologic response: 100% (11/11)	In-hospital mortality: 27.3%	[65]
	Retrospective cohort study	Pneumonia, BSI, cIAI, osteomyelitis, UTI	Clinical success: 73% (40/55) overall Monotherapy: 100% (3/3) Combination: 77% (37/52) Microbiologic eradication: 78% (43/55, documented or presumed) Monotherapy: 2 documented, 1 presumed, 0 failures Combination: 29 documented, 11 presumed, 12 failures	Infection-related mortality: 25%	[66]
Polymyxins	Randomized controlled trial (OVERCOME)	Pneumonia and BSI caused by XDR *A. baumannii*, XDR *P. aeruginosa*, or CRE	Overall clinical failure: 65% (119/184) for colistin monotherapy; 58% (110/190) for colistin–meropenem Overall microbiological cure: 65% (106/163) for colistin monotherapy; 60% (103/171) for colistin–meropenem	28-d: 43% for colistin monotherapy; 37% for colistin–meropenem	[67]
	Randomized controlled trial (AIDA)	Pneumonia and BSIs	D 14 treatment failure: 79% (156/198) for colistin monotherapy; 73% (152/208) for colistin–meropenem	14-d: 32% (colistin monotherapy); 34% (colistin–meropenem) 28-d: 43% (colistin monotherapy); 45% (colistin–meropenem)	[68]
Sulbactam-Durlobactam	Phase III randomized controlled trial (ATTACK)	HABP, VABP, BSI	Clinical cure at TOC: 62% (39/63) for sulbactam-durlobactam; 40% (25/62) for colistin Sustained clinical cure at late follow-up: 43% (27/63) for sulbactam-durlobactam; 31% (19/62) for colistin Microbiological response at end of therapy: 86% (54/63) for sulbactam-durlobactam; 61% (38/62) for colistin Microbiological response at TOC: 68% (43/63) for sulbactam-durlobactam; 42% (26/62) for colistin	14-d: 6% for sulbactam-durlobactam; 19% for colistin 28-d: 19.0% for sulbactam-durlobactam; 32.3% for colistin	[58]
Tigecycline	Retrospective cohort study	HAP due to CROs	Clinical success: 34.3% (59/173)	14-d: 26.6% 28-d: 39.3%	[69]

Abbreviations: BAT, best available therapy; BSI, bloodstream infections; cIAI, complicated intra-abdominal infection; CRAB, carbapenem-resistant *Acinetobacter baumanni;* CRE, carbapenem-resistant Enterobacterales; CRO, carbapenem-resistant organism; HAP, hospital-acquired pneumonia; HABP, hospital-acquired bacterial pneumonia; HCAP, healthcare-associated pneumonia; UTIs, urinary tract infections; VAP, ventilator-associated pneumonia; VABP, ventilator-associated bacterial pneumonia; XDR, extensively drug-resistant; XDRAB, extensively drug-resistant *A. baumannii.*

Polymyxins, including colistin and polymyxin B, are still frequently used to manage severe CRAB infections in some regions, particularly in ICUs. These agents demonstrate broad activity against MDR strains, but their clinical utility is limited by nephrotoxicity, neurotoxicity, and the emergence of polymyxin-resistant strains [37, 70–73]. Although combination regimens involving polymyxins, such as colistin–carbapenem or colistin–sulbactam, have shown improved microbiological cure rates compared with monotherapy, the evidence remains inconsistent, and treatment outcomes vary depending on local resistance patterns and access to resources [9, 68, 74]. The Clinical and Laboratory Standards Institute (CLSI) has eliminated the susceptible interpretive category for polymyxins, citing the inability of these agents to achieve stasis in pneumonia models and their inconsistent pharmacokinetics, which often result in suboptimal drug exposure [75]. Additionally, pharmacokinetic/pharmacodynamic (PK/PD) studies have demonstrated that polymyxins fail to achieve bacterial stasis in lung infection models, even at the highest tolerated doses, further questioning their role in the treatment of CRAB pneumonia [76]. Given these limitations, reliance on polymyxins, particularly as monotherapy, should be approached with caution, and alternative agents should be prioritized when available. The OVERCOME trial compared the efficacy of colistin monotherapy to colistin–meropenem combination therapy for pneumonia and BSIs caused by extensively drug-resistant Gram-negative bacteria, of which 78% were due to *A. baumannii*. This randomized trial concluded that there was no significant difference in 28-day mortality between the monotherapy and combination therapy groups (43% vs 37%, respectively; *P* = .17). Notably, while numerical trends suggested potential benefits of combination therapy for infections caused by carbapenem-resistant Enterobacterales (CRE) and carbapenem-resistant *Pseudomonas aeruginosa* (CRPA), no such benefit was observed for CRAB infections [67]. Another randomized trial, the AIDA trial, also did not find superiority of colistin–meropenem combination therapy over colistin monotherapy, with 28-day mortality rates of 43% and 45%, respectively [68]. The high mortality rates observed in both studies underscore that colistin and colistin–carbapenem combinations are not effective treatment options for CRAB infections.

However, other studies suggest that different combination regimens may reduce the emergence of resistance and improve outcomes in severely ill patients [74, 77, 78]. For example, high-dose colistin combinations with fosfomycin or inhaled colistin have been associated with improved clinical outcomes in patients with nosocomial pneumonia caused by CRAB [79].

Minocycline has gained renewed attention as a therapeutic option for CRAB infections, particularly in combination with other agents. A systematic review of 10 studies, including 9 retrospective case series and one single-center trial, found that 223 of 268 patients (83.2%) received a minocycline-based regimen, with 91.7% of these patients receiving minocycline in combination with other agents, most commonly colistin or carbapenems [80]. The pooled clinical and microbiological success rates were 72.6% and 60.2%, respectively, and the mortality rate was 20.9% among patients with available outcome data [80]. Minocycline is generally well tolerated in critically ill patients, with adverse events reported infrequently and typically mild. In most studies, patients received concurrent nephrotoxic agents, limiting the ability to directly attribute toxicity to minocycline. While dose adjustment is not routinely required in renal impairment, caution may be warranted in select cases. Recent pharmacokinetic and pharmacodynamic analyses have led to a reduction in the CLSI susceptibility breakpoint from 4 to 1 mg/L, based on a higher dosage of 200 mg every 12 hours, to optimize target attainment and improve clinical outcomes [80].

Tigecycline has shown efficacy when used in combination regimens for CRAB infections but is less effective in BSI and UTI due to suboptimal PK/PD properties [78, 81]. High-dose regimens have been proposed to optimize tigecycline's PK/PD profile, yet resistance mechanisms such as efflux pump overexpression limit its therapeutic potential [37]. Moreover, high-dose tigecycline is associated with an increased risk of gastrointestinal adverse effects, including nausea and vomiting [82], which may impact tolerability, particularly in critically ill patients. Despite limited efficacy in BSI, tigecycline has shown promise in combination therapies, particularly with colistin or fosfomycin, to enhance its clinical effectiveness in severe CRAB infections [79].

Eravacycline, a newer fluorocycline, is a tetracycline-class option for CRAB. In vitro and surveillance work generally shows lower MICs and broader activity than tigecycline across prevalent OXA-23 backgrounds, with an MIC_90_ of 2 µg/mL and retaining activity against some tigecycline-resistant isolates [46, 47]. Early clinical experience—mainly in critically ill patients with HAP/VAP—suggests encouraging responses and good tolerability, particularly in combination regimens [44, 48, 83]. A single-center observational study of 65 ICU patients with CRAB pneumonia indicated similar effectiveness versus tigecycline and numerically lower 30-day mortality, while early (≤72 hours) initiation in immunocompromised patients was associated with reduced risk of failure and infection recurrence in a multicenter observational study [84, 85]. However, no randomized trials of eravacycline for CRAB infections have been conducted. Where tetracycline-class therapy is considered, eravacycline is an alternative to tigecycline and can be used as part of combination therapy, guided by local susceptibility patterns and stewardship principles [48, 84, 85].

Similarly, high-dose ampicillin–sulbactam remains an option for CRAB infections due to sulbactam's intrinsic activity against *Acinetobacter* species. Resistance mediated by upregulated β-lactamases or mutations in PBPs, significantly reduces its effectiveness [37]. High-dose ampicillin–sulbactam, defined as regimens delivering ∼27 g per day (equivalent to 9 g of sulbactam), is typically administered either by continuous infusion or by extended infusion in order to achieve sustained saturation of PBP targets [86]. High-dose sulbactam combinations, particularly with tigecycline, have been shown to improve clinical outcomes in MDR/XDR *Acinetobacter* infections [87].

Cefiderocol, a novel siderophore cephalosporin, has emerged as a promising agent against carbapenem-resistant strains, however, issues with AST, including heteroresistance, variability in testing methods, and challenges in breakpoint interpretation, pose significant barriers to its widespread adoption [33, 40, 88]. These challenges complicate the accurate assessment of cefiderocol's efficacy in both laboratory and clinical settings, potentially affecting treatment outcomes. The *CREDIBLE-CR* study evaluated cefiderocol's efficacy in serious infections caused by MDR/XDR Gram-negative bacteria. While cefiderocol demonstrated comparable clinical and microbiological outcomes to best available therapy (BAT), a numerically higher all-cause mortality rate was observed in the cefiderocol-treated arm, particularly in patients with CRAB infections, raising concerns about its role in treating CRAB [40]. Notably, among the 34 cefiderocol-treated patients who died, 21 had CRAB infections, highlighting a potential limitation of cefiderocol in this pathogen group [40].

The APEKS-NP trial assessed cefiderocol for nosocomial pneumonia and demonstrated noninferiority to high-dose, prolonged-infusion meropenem for Gram-negative pneumonia. However, subgroup analyses showed no clinical benefit over meropenem in *A. baumannii* pneumonia or for meropenem-resistant pathogens, reinforcing uncertainties regarding cefiderocol's effectiveness against CRAB [64]. Given the limitations observed in these studies, cefiderocol's role in treating CRAB infections remains uncertain, and combination therapy strategies are being explored. Some studies suggest that pairing cefiderocol with other agents, such as fosfomycin, may enhance its efficacy [79, 88].

The European Society of Clinical Microbiology and Infectious Diseases (ESCMID) 2022 guidelines recommend ampicillin–sulbactam for sulbactam-susceptible CRAB infections but do not specify a preferred agent for sulbactam-resistant strains, instead suggesting polymyxins or high-dose tigecycline if active in vitro. Additionally, ESCMID conditionally recommends against cefiderocol for CRAB due to concerns about increased mortality in clinical trials. The guidelines also discourage polymyxin–meropenem and polymyxin–rifampin combination therapy, while suggesting that combination regimen with 2 in vitro active agents may be beneficial for severe or high-risk CRAB infections [89].

The Infectious Diseases Society of America (IDSA) 2024 guidance document recommends SUL/DUR in combination with a carbapenem as the preferred first-line regimen for CRAB infections. When SUL/DUR is unavailable, high-dose ampicillin–sulbactam in combination with at least one other agent (eg, polymyxin B, minocycline, tigecycline, or cefiderocol) is suggested as an alternative [38].

## SUL/DUR: A NEW THERAPY

The emergence of CRAB and lack of effective therapies has created an urgent need for new approaches to management CRAB infections. SUL/DUR, a novel β-lactam/β-lactamase inhibitor combination, represents a significant breakthrough in addressing this challenge. Approved by the U.S. Food and Drug Administration (FDA) in 2023 for the treatment of hospital-acquired bacterial pneumonia (HABP) and ventilator-associated bacterial pneumonia (VABP) in patients 18 years of age and older caused by the *Acinetobacter baumannii–calcoaceticus* complex (ABC) [90], SUL/DUR aims to restore the bactericidal activity of sulbactam against resistant strains of *A. baumannii* [91]. Sulbactam's β-lactam activity is complemented by durlobactam, a diazabicyclooctane (DBO) β-lactamase inhibitor that prevents enzymatic degradation. Together, these agents provide a dual-action mechanism, combining sulbactam's antibacterial properties with durlobactam's inhibition of resistance-conferring β-lactamases [91]. Administered as a copackaged product containing 1 g of sulbactam and 1 g of durlobactam via IV infusion over 3 hours every 6 hours, the dosing regimen is adjusted based on renal function, ensuring optimal efficacy and safety across diverse patient populations [90].

SUL/DUR demonstrates potent in vitro activity against ABC, including highly resistant strains [92–94]. A global study of 5032 isolates collected from 33 countries between 2016 and 2021 showed that adding durlobactam reduced sulbactam's MIC_90_ from 64 to 2 μg/mL, with 98.3% susceptibility at the FDA breakpoint of ≤4 μg/mL [95]. The activity was consistent across geographic regions, years, and infection sources, with >96% susceptibility observed in carbapenem-nonsusceptible, colistin-resistant, MDR, and XDR isolates [57, 95] (Table 3).

**Table 3.** In Vitro Activities of SUL/DUR and Comparator Antimicrobial Agents Tested Against 5032 Clinical Isolates of *A. Baumannii*–calcoaceticus Complex Species Collected Globally From 2016 to 2021^a^

Species (No. of Isolates)	Antimicrobial Agent	MIC50 (µg/mL)	MIC90 (µg/mL)	Range (µg/mL)	Susceptible (%)	Intermediate (%)	Resistant (%)
All isolates (5032)^b^	SUL/DUR^c^	1	2	≤0.03–>64	98.3	NA	1.7
	Sulbactam^d^	8	64	0.25–>64	46.9	8.0	45.1
	Cefepime	16	>16	≤0.12–>16	44.6	7.9	47.4
	Imipenem	8	>64	≤0.03–>64	48.9	0.6	50.5
	Meropenem	16	>64	≤0.03–>64	47.9	1.1	51.0
	Amikacin	4	>64	≤0.5–>64	58.6	3.3	38.1
	Ciprofloxacin	>4	>4	≤0.12–>4	44.4	0.7	54.9
	Colistin^e^	0.5	1	≤0.25–>8	NA	95.9	4.1
	Minocycline	0.5	16	≤0.12–>16	78.3	10.1	11.6
	Tigecycline^f^	0.5	2	0.03–32	NA	NA	NA
*A. baumannii* (4038)	Sulbactam-durlobactam	1	2	≤0.03–>64	98	NA	2.0
	Sulbactam	16	64	0.25–>64	36.5	8.9	54.6
	Cefepime	>16	>16	≤0.12–>16	33.6	8.8	57.6
	Imipenem	32	>64	≤0.03–>64	37.7	0.6	61.6
	Meropenem	64	>64	≤0.03–>64	36.6	1.2	62.3
	Amikacin	32	>64	≤0.5–>64	49.5	3.8	46.6
	Ciprofloxacin	>4	>4	≤0.12–>4	32.7	0.7	66.6
	Colistin	0.5	1	≤0.25–>8	NA	95.1	4.9
	Minocycline	1	16	≤0.12–16	73.3	12.4	14.4
	Tigecycline^f^	0.5	2	0.03–32	NA	NA	NA

^a^ABC, *Acinetobacter baumannii–calcoaceticus* complex; NA, not available.

^b^There were 4 isolates of non-identified *Acinetobacter spp.* and 1 isolate of *Acinetobacter dijkshoorniae* that are included in the total data set but not divided out individually in the table.

^c^SUL/DUR MICs were interpreted using the preliminary MIC breakpoints of ≤4 µg/mL (susceptible) and ≥8 µg/mL (resistant).

^d^Sulbactam MICs were interpreted using the sulbactam component of CLSI M100 (2021) ampicillin–sulbactam MIC breakpoints (≤8/4 [susceptible], 16/8 [intermediate], and ≥32/16 [resistant]) given that sulbactam is well established to comprise the active component of the combination for *Acinetobacter spp.*

^e^CLSI M100 (2021) lists only intermediate and resistant MIC breakpoints for colistin tested against *Acinetobacter spp.*

^f^MIC interpretative criteria are not published by CLSI M100 (2021) for tigecycline tested against *Acinetobacter spp*.

Table adapted from [95].

### Mechanism of Action

The activity of SUL/DUR lies in its synergistic mechanism of action. Sulbactam, originally developed as a β-lactamase inhibitor, exhibits bactericidal activity against *Acinetobacter* species by targeting PBPs, particularly PBP1 and PBP3; these proteins are essential for bacterial cell wall synthesis, and their inhibition results in cell lysis [35]. Sulbactam's direct antibacterial activity against *A. baumannii* makes it a cornerstone for treating carbapenem-resistant infections. However, sulbactam's efficacy is limited due to degradation by various β-lactamases, including class D OXA-type enzymes [96].

Durlobactam protects sulbactam by inhibiting β-lactamases that degrade sulbactam, particularly OXA carbapenemases, which are prevalent in *A. baumannii*. Durlobactam's structural modifications, such as enhanced reactivity and superior binding to class A, C, and D β-lactamases, distinguish it from earlier DBO inhibitors like avibactam. Its reversible mechanism involves active site carbamoylation, which restores sulbactam's activity even against MDR and XDR isolates. Studies demonstrate durlobactam's ability to achieve greater potency, reducing resistance rates to as low as 2.3% compared with cefoperazone/sulbactam (46.3%), a combination frequently used in some regions for *A. baumannii* infections despite cefoperazone lacking β-lactamase inhibitory activity [96].

### Pharmacokinetics and Pharmacodynamics (PK/PD)

The PK/PD of SUL/DUR are informed by extensive studies on its individual components. Sulbactam demonstrates optimal antibacterial activity (≥1-log₁₀ CFU reduction) when unbound concentrations remain above the minimum inhibitory concentration (MIC) for approximately 25–50% of the dosing interval (fT > MIC), depending on the infection site and model. Durlobactam requires an area under the curve (AUC)/MIC ratio of 10 for effective β-lactamase inhibition. These PK/PD parameters have been validated in preclinical models, including neutropenic murine thigh and lung infection models, as well as hollow-fiber infection systems. In these models, SUL/DUR achieved bactericidal effects (≥1-log₁₀ CFU reduction) within 24 hours when sulbactam exposures exceeded 32.9% fT > MIC in the thigh model and 24.5% fT > MIC in the lung model [97].

Recent population PK analyses integrating Phase 1, 2, and 3 clinical data further refined the understanding of SUL/DUR's disposition, highlighting its linear pharmacokinetics and renal elimination as a primary clearance pathway [98]. A 4-compartment model with separate renal and nonrenal clearance estimates for sulbactam and durlobactam confirmed that renal function is the most clinically significant covariate affecting drug exposure, necessitating dose adjustments in patients with impaired renal clearance. Additionally, epithelial lining fluid (ELF) penetration studies demonstrated that sulbactam achieves ELF concentrations ∼86% of the corresponding free plasma concentration, whereas durlobactam attains an ELF penetration ratio of 41.3%. These findings suggest adequate pulmonary exposure, supporting the use of SUL/DUR for pneumonia caused by CRAB.

Furthermore, hemodialysis studies revealed that intermittent dialysis significantly reduces SUL/DUR exposure, leading to an ∼30% decrease in 24-hour AUC when dialysis is initiated shortly after dosing. These findings highlight the need for careful dose optimization in patients undergoing renal replacement therapy [98]

### Clinical Efficacy

The clinical efficacy and safety of SUL/DUR were demonstrated in the pivotal Phase III ATTACK trial, a global, multicenter, randomized trial. This trial evaluated SUL/DUR against colistin in patients with confirmed CRAB infections, including HABP, VABP, and BSI, with both treatment arms receiving imipenem–cilastatin as background therapy to provide additional coverage for potential coinfecting pathogens. The trial's primary endpoint, 28-day all-cause mortality, established that SUL/DUR was noninferior to colistin, with 12 of 63 patients (19.0%) in the SUL/DUR group dying within 28 days compared with 20 of 62 patients (32.3%) in the colistin group. This finding underscores SUL/DUR's potential to improve survival in critically ill patients, with a treatment difference of −13.2% (95% CI: −30.0% to 3.5%) within the prespecified noninferiority margin [58].

Secondary endpoints also highlighted SUL/DUR's clinical advantages. Clinical cure rates at the test of cure (TOC) were significantly higher in the SUL/DUR group, with 61.9% of patients achieving a cure compared with 40.3% in the colistin arm, reflecting a treatment difference of 21.6% (95% CI: 2.9%, 40.3%). Microbiological eradication was similarly favorable, achieved in 68.3% of SUL/DUR-treated patients versus 41.9% in the colistin group, demonstrating a treatment difference of 26.3% (95% CI: 7.9%, 44.7%). These results consistently favored SUL/DUR across all subpopulations evaluated, including critically ill patients with varying degrees of renal impairment and complex comorbidities [58].

In addition to clinical outcomes, microbiological analyses in the ATTACK trial provided valuable insights. Among the baseline ABC isolates, 96% were carbapenem-resistant, and 85% were extensively drug-resistant (XDR). Despite this high level of resistance, 95.4% of sulbactam-nonsusceptible isolates became susceptible when combined with durlobactam, achieving MIC values of ≤4 µg/mL. Notably, even in the presence of the most resistant isolates, SUL/DUR demonstrated a potent microbiological response, supporting its use in diverse global settings. Importantly, only one patient randomized to SUL/DUR developed on-therapy resistance, indicating a low propensity for resistance emergence during treatment [99].

### Safety

The trial's safety data further emphasized SUL/DUR's superiority over colistin. Nephrotoxicity, a major concern with polymyxin-based therapies, was significantly lower in the SUL/DUR group (13.2% vs 27.6%; *P* < .001). This reduction is particularly important for the critically ill population, where renal dysfunction is common due to the use of multiple nephrotoxic agents and underlying conditions. The incidence of treatment-emergent adverse events (TEAEs) was high across both groups, as expected in this severely ill cohort, but the overall profile favored SUL/DUR. TEAEs occurred in 88% of patients in the SUL/DUR group compared with 95% in the colistin group, and severe TEAEs were also less frequent with SUL/DUR [58].

Notably, SUL/DUR demonstrated lower rates of emergent infections and superinfections (1% vs 29%) and *Clostridioides difficile* infections (7% vs 19%) compared with colistin. Treatment-related adverse events leading to discontinuation of therapy were also less frequent in the SUL/DUR group (11% vs 16%). The most common drug-related adverse events with SUL/DUR were mild, including diarrhea and pneumonia, each reported in only 2% of patients. One case of anaphylactic shock was reported in the SUL/DUR arm, leading to treatment discontinuation [58].

The question of whether SUL/DUR should be combined with a carbapenem, such as imipenem or meropenem, has been explored in some studies. In vitro studies have further investigated the impact of carbapenem combination therapy. Iovleva et al [100]. demonstrated that the addition of imipenem lowered SUL/DUR MICs by 1–2 dilutions in 85%–96% of tested isolates, potentially enhancing its activity against CRAB. Furthermore, O’Donnell et al [97]. evaluated SUL/DUR in dynamic in vitro models, including hollow-fiber infection systems, and found that while SUL/DUR alone achieved bactericidal activity, the addition of imipenem enhanced bacterial killing in some isolates, suggesting a possible synergistic effect. Building on these findings, Veeraraghavan et al [101]. used static time-kill and molecular modeling studies to demonstrate synergy between imipenem and sulbactam in the presence of durlobactam. Their results showed ≥2-log₁₀ CFU/mL reductions with the triple combination and suggested that enhanced killing was due to complementary PBP binding and durlobactam-mediated protection of both agents from OXA carbapenemases. These results support the rationale for further clinical investigation into combination therapy, particularly in patients with high bacterial burdens or borderline susceptibility. However, whether these in vitro findings translate into improved clinical outcomes remains uncertain and requires additional study.

### Role in Clinical Practice

SUL/DUR is now recommended as a first-line treatment option for CRAB infections, particularly in critically ill patients with HABP, VABP, or BSIs [38]. The 2024 IDSA guidance endorses SUL/DUR over traditional therapies such as colistin and cefiderocol due to its superior safety and efficacy profile. Notably, the IDSA highlights its significantly lower nephrotoxicity compared with colistin, alongside higher rates of microbiological eradication [38]. In ICU settings, SUL/DUR is particularly advantageous for patients with complex infections, including VAP. Its predictable pharmacokinetics and strong clinical trial data, such as findings from the pivotal ATTACK trial, support its inclusion in hospital formularies for centers that care for patients infected with CRAB, ensuring its availability for high-risk populations [38].

While resistance to SUL/DUR remains uncommon, some mechanisms have been identified, including PBP3 mutations (eg, T526S and G523 V), which reduce sulbactam binding, and metallo-β-lactamases (MBLs), which durlobactam does not inhibit [102].

Looking ahead, further research on combination therapies and real-world implementation will clarify SUL/DUR's role in combating CRAB infections. As resistance patterns continue to evolve, SUL/DUR remains an essential tool in managing these challenging infections, providing a critical option for improving outcomes in vulnerable patient populations [38].

## CONCLUSION

Looking forward, the integration of robust surveillance, rapid diagnostics, and antimicrobial stewardship programs will be essential to optimize the use of SUL/DUR and mitigate the impact of CRAB infections worldwide. However, resistance development remains an ongoing threat, underscoring the need for continued innovation.

Several new drugs in the pipeline also offer hope for combating CRAB infections. Non-β-lactam–β-lactamase inhibitor combinations, such as WCK 4234 plus meropenem (WCK-5999) and LN-1-255 plus meropenem or imipenem, are being evaluated for their potential to enhance the efficacy of existing treatments [103, 104]. Additionally, zosurabalpin, a narrow-spectrum antibiotic targeting the LptB2FGC complex in the lipopolysaccharide (LPS) transport system of *A. baumannii*, has shown promising results. Preclinical studies highlight its efficacy in mouse infection models and in vitro activity against CRAB isolates, along with favorable safety and pharmacokinetic profiles. Currently in clinical trials, zosurabalpin represents a novel approach to treating invasive CRAB infections [105].

As these new therapies advance through clinical development, they will play an essential role in expanding the arsenal of treatment options for CRAB. Continued innovation, combined with optimized use of current therapies like SUL/DUR, will be critical to addressing the growing threat of carbapenem-resistant pathogens and improving outcomes for vulnerable patient populations.

CRAB remains a formidable global health challenge, driven by its high resistance rates, limited treatment options, and significant mortality burden. The introduction of SUL/DUR marks a promising advancement, addressing the limitations of existing therapies through its synergistic mechanism of action and favorable clinical outcomes, as demonstrated in the ATTACK trial. Despite the emergence of resistance in some strains, SUL/DUR's efficacy and safety profile establish it as a critical therapeutic option in the fight against CRAB.
